# Simple nontoxic goiter in the portrayal of Saints: a magnificent representation in one piece of art

**DOI:** 10.1007/s40618-023-02097-1

**Published:** 2023-04-28

**Authors:** A. Berlińska, R. Świątkowska-Stodulska

**Affiliations:** Department of Endocrinology and Internal Medicine, Faculty of Medicine, Medical University of Gdańsk, Gdańsk, Poland

A painting entitled *Three Saints* or *Three Holy Virgins* is a fine example of medieval art and dates back to the fifteenth century. Based on the characteristic presentation, it is likely that two of the models suffered from endemic goiter dependent on iodine deficiency. According to official information from the Diocesan Museum of Sandomierz [1], where the painting is displayed, the unknown author of the masterpiece worked in Lesser Poland, a region located in the southern part of the country, close to the Carpathian Mountains, an environment traditionally associated with low availability of iodine [2]. In addition to iodine deficiency, which is the most common and widespread cause of endemic goiter, pregnancy, dyshormonogenesis, postpartum thyroiditis, and autoimmune thyroid disorders could trigger the development of goiters that we can now admire immortalized as works of art [3–5]. In Poland, first iodine supplementation attempts started in the 1930s in Lesser Poland and became countrywide in 1997 [2].

Although it is uncertain which Saints are displayed in the painting, with Martha, Agnes, and Clare being the most common assumptions, the artist managed to capture humanity in Saints, even if unintentionally. Perhaps if the neck of the rightmost model was not covered by a veil, we would be able to see yet another magnificent goiter (Fig. 1).

**Fig. 1:**
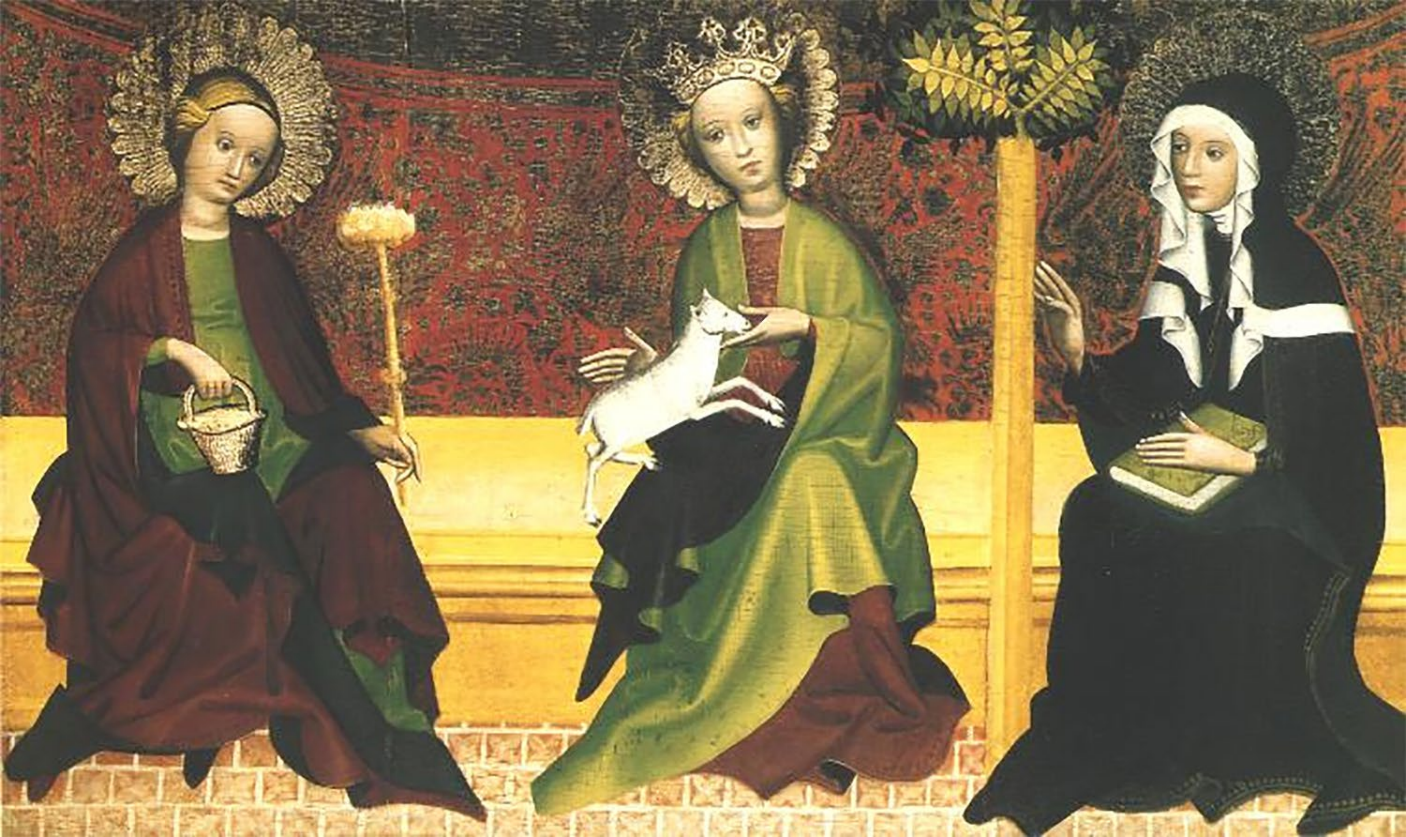
Unknown Polish Author (XV century): *Three Saints* or *Three Holy Virgins,* tempera painting on wooden panels, 48,5 × 79,5 cm, Muzeum Diecezjalne w Sandomierzu (Diocesan Museum of Sandomierz), Sandomierz, Poland. Image source: Długosz House, Public domain, via Wikimedia Commons

## Data Availability

Data sharing not applicable to this article as no datasets were generated or analysed during the current study.
